# Real-time monitoring of subsurface microbial metabolism with graphite electrodes

**DOI:** 10.3389/fmicb.2014.00621

**Published:** 2014-11-21

**Authors:** Colin Wardman, Kelly P. Nevin, Derek R. Lovley

**Affiliations:** Department of Microbiology, University of Massachusetts AmherstAmherst, MA, USA

**Keywords:** subsurface sediments, microbial activity, anaerobic metabolism, electromicrobiology, aquatic sediments, biogeochemistry

## Abstract

Monitoring *in situ* microbial activity in anoxic submerged soils and aquatic sediments can be labor intensive and technically difficult, especially in dynamic environments in which a record of changes in microbial activity over time is desired. Microbial fuel cell concepts have previously been adapted to detect changes in the availability of relatively high concentrations of organic compounds in waste water but, in most soils and sediments, rates of microbial activity are not linked to the concentrations of labile substrates, but rather to the turnover rates of the substrate pools with steady state concentrations in the nM–μM range. In order to determine whether levels of current produced at a graphite anode would correspond to the rates of microbial metabolism in anoxic sediments, small graphite anodes were inserted in sediment cores and connected to graphite brush cathodes in the overlying water. Currents produced were compared with the rates of [2-^14^C]-acetate metabolism. There was a direct correlation between current production and the rate that [2-^14^C]-acetate was metabolized to ^14^CO_2_ and ^14^CH_4_ in sediments in which Fe(III) reduction, sulfate reduction, or methane production was the predominant terminal electron-accepting process. At comparable acetate turnover rates, currents were higher in the sediments in which sulfate-reduction or Fe(III) reduction predominated than in methanogenic sediments. This was attributed to reduced products (Fe(II), sulfide) produced at distance from the anode contributing to current production in addition to the current that was produced from microbial oxidation of organic substrates with electron transfer to the anode surface in all three sediment types. The results demonstrate that inexpensive graphite electrodes may provide a simple strategy for real-time monitoring of microbial activity in a diversity of anoxic soils and sediments.

## Introduction

Anaerobic microbial processes play an important role in the biogeochemistry of submerged soils and aquatic sediments, as well as in deeper subsurface environments (Yavitt et al., 1987; Canfield et al., 1993; Chapelle, 1993; Lovley and Chapelle, 1995; Liesack et al., 2000). Which anaerobic process predominates within a given environment can be simply determined from measurements of steady-state H_2_ concentrations (Lovley and Goodwin, 1988; Lovley et al., 1994; Chapelle et al., 1997). However, assessing the rates of anaerobic processes has proven to be more difficult.

Most strategies for estimating rates of anaerobic microbial metabolism involve incubating soil/sediment subsamples. This approach typically requires sophisticated analytical techniques for analyzing the products of microbial metabolism and, in some instances, can dramatically change rates of microbial activity (Chapelle and Lovley, 1990; Phelps et al., 1994). The labor and expense of such measurements often negate the possibility of making detailed time series of microbial rate measurements that are required for studies on the response of microbial activity to seasonal changes or environmental disturbances, such as the introduction of contaminants.

Early studies noted a correlation between the availability of organic substrates and current production in microbial fuel cells (Bond and Lovley, 2003) and a number of studies have demonstrated that the current output of microbial fuel cells can be used to measure the concentrations of defined substrates added to water or as an estimate of the amount of microbially degradable organic matter in wastewater (Kumlanghan et al., 2007; Tront et al., 2008; Di Lorenzo et al., 2009; Williams et al., 2010; Zhang and Angelidaki, 2011). With the exception of one study that evaluated the abundance of acetate in groundwater amended with acetate for *in situ* uranium bioremediation (Williams et al., 2010), microbial fuel cell technology for estimating substrate concentrations has relied on laboratory-scale devices that would require that samples be taken from the environment for analysis. The substrate concentrations evaluated with this sensing technology has been in the mM range whereas the concentrations of readily degradable organic substrates in most anoxic soils and sediments are in the μM range or less.

Furthermore, although microbial activity may be directly linked to the concentrations of readily degradable organic substrates in artificial environments, such as wastewater digesters, or when organic substrates are added to promote groundwater bioremediation, there is not a clear link between the concentrations of readily measured substrates and microbial activity in most anoxic soils and sediments. In fact, the pool sizes of readily degradable organic substrates such as fermentable sugars and amino acids, as well as acetate and H_2_, the prime intermediates for anaerobic respiration, are uniformly low regardless of the rates of metabolism. Rates of microbial metabolism are reflected in the turnover rates of these substrate pools, not their concentrations. For example, this is clearly evident with the fermentation product H_2_. The H_2_-consuming microbial community rapidly adjusts to variations in the rate of H_2_ production and maintains the H_2_ pool at concentrations that are just high enough that H_2_ oxidation is still thermodynamically favorable with the most electro-positive electron acceptor that is available for H_2_ oxidation (Lovley and Goodwin, 1988). Therefore, environments that differ in rates of H_2_ production even by an order of magnitude will have approximately the same H_2_ concentrations if the same terminal electron accepting process predominates. The difference in the H_2_ production rates will be reflected in the size of the H_2_-consuming microbial community, the environment with a 10-fold higher rate of H_2_ production will have a correspondingly higher biomass of H_2_-consuming microorganisms coupled with a correspondingly higher rate of the reduction of terminal electron acceptors (Lovley and Goodwin, 1988). Similar considerations apply to other substrates.

**Figure 1:**
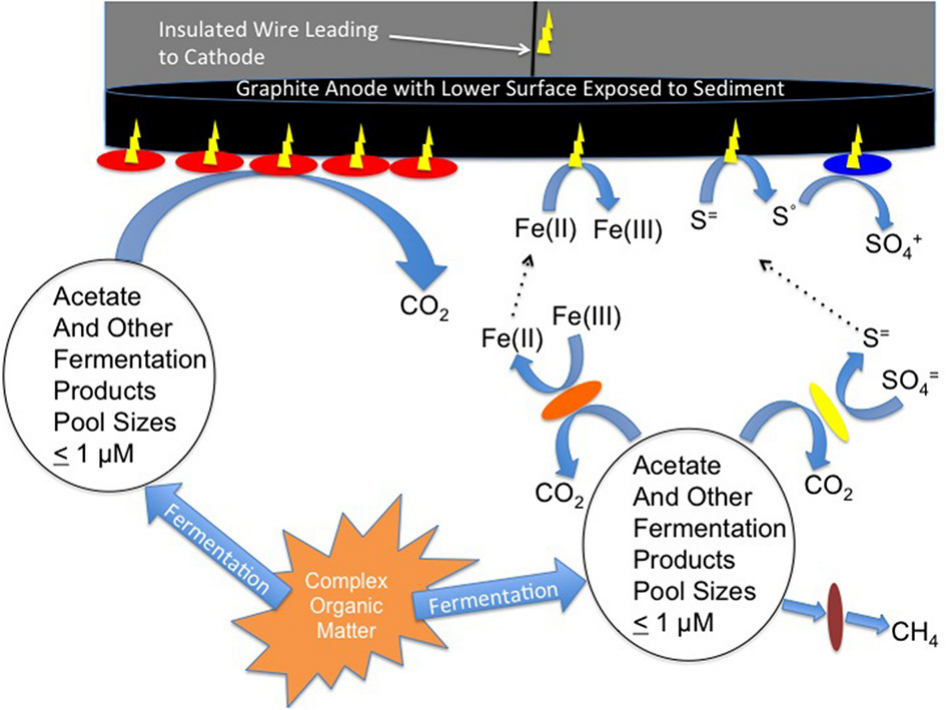
**Model for current production with microbial activity sensors**. Acetate and other fermentation products produced from the hydrolysis and fermentation of particulate matter serve as electron donors for microbial current production at the anode surface. At distance from the anode these fermentation products are electron donors for methane production, sulfate reduction or Fe(III) reduction. Methane is not reactive with the anode, but Fe(II) and sulfide can be abiotically oxidized at the anode. Elemental sulfur produced from the oxidation of sulfide can serve as an electron donor for additional microbially catalyzed current production.

An anode introduced into an anoxic sediment is simply an alternative to other electron acceptors for anaerobic respiration such as Fe(III), sulfate, or carbon dioxide. Therefore, the amount of current generated from anodes can also be expected to be related to the turnover rate of electron donors that can contribute to current production. Acetate, is typically the most important intermediary in carbon and electron flow in anoxic sediments (Lovley and Chapelle, 1995) and acetate-oxidizing microorganisms often predominate on current-harvesting electrodes inserted in anoxic soils and sediments (Lovley, 2006; Lovley et al., 2011). The production and consumption of other organic substrates, as well as H_2_, may also contribute to current production (Figure 1). The rate that all of these potential electron donors are produced from complex organic material near an anode inserted in anoxic soils and sediments should be reflected in the amount of current production. If so, there should be a direct correlation between rates of acetate turnover and current production in sediments with different rates of microbial metabolism because changes in the rate of organic matter metabolism will be accompanied by a corresponding change in the acetate turnover rate.

Other potential electron donors for current generation are reduced products of anaerobic respiration that takes place at distance from the anode (Reimers et al., 2001). These include Fe(II), the product of Fe(III) reduction, and sulfide, the product of sulfate reduction. Both Fe(II) and sulfide can diffuse through sediments and abiotically donate electrons to electrodes (Figure 1). Furthermore, the elemental sulfur produced at the anode surface from the abiotic oxidation of sulfide can serve as an electron donor for current production by microorganisms, such as *Desulfobulbus* (Holmes et al., 2004) and *Desulfuromonas* (Zhang et al., 2014) species.

A previous study demonstrated that, even in an organic poor subsurface soil, the indigenous rate of production of fermentation intermediates was sufficient to yield low but detectable currents (0.05–0.2 mA/m^2^) from graphite electrodes deployed in the subsurface and connected through a resistor to graphite cathodes at the ground surface (Williams et al., 2010). Currents were also detectable when poised graphite electrodes were deployed in Artic peat soils (Friedman et al., 2012). In the later studies, the electrodes were poised to specifically monitor the activity of Fe(III)- and humic-reducing microorganisms, and thus required a poteniostat, which made the monitoring system more complicated than the simple, anode-resister-cathode configuration. Instability of reference electrodes is likely to limit the long-term applicability of the poised anode approach. There were changes in currents produced within the Artic peats, some of which corresponded with diurnal temperature changes, suggesting that the current produced might be correlated with microbial activity (Friedman et al., 2012). However, no independent measurements of microbial activity were made.

Here we report on a simple anode-resister-cathode system for monitoring the natural activity of a diversity of microorganisms. We demonstrate a direct correlation between current production and rates of microbial activity as determined by the turnover of tracer [2-^14^C]-acetate in sediments in which Fe(III) reduction, sulfate reduction, or methane production was the predominate terminal electron-accepting process.

## Methods and materials

### Sediment sources

In order to evaluate the relationship between current production and microbial activity in a diversity of sediments, sediments were collected from sites at which Fe(III) reduction, sulfate reduction, or methane production was the predominant terminal electron accepting processes. Sediments in which Fe(III) reduction was the predominant terminal electron accepting process were collected from the previously described (Anderson et al., 2003; Williams et al., 2011) uranium-contaminated aquifer located in Rifle, CO. As previously described (Barlett et al., 2012), subsurface sediments were collected with a backhoe, stored in five gallon buckets, shipped to the laboratory at the University of Massachusetts, and stored at 15°C.

Sediments in which sulfate reduction was the predominant terminal electron accepting process were collected from the previously described (Broadaway and Hannigan, 2012) study site in Nantucket, MA. At low tide, in the center of the salt marsh (water level 0.25 m), the oxidized zone (top 3–5 cm) was removed from the sediment in place and the underlying sediment depth of approximately 5 to 25 cm was collected by shovel, placed into mason jars, sealed without a headspace, and transported back to the laboratory. The sediments were stored at 15°C.

Sediments in which methane production was the predominant terminal electron accepting process were collected from Puffers Pond, Amherst, MA. Sediments were collected from areas where active methane gas ebullition was observed when a rod was inserted into the sediment. The water depth at sampling locations was 0.1 to 0.25 m. As described above for the Nantucket site sediments, the overlying oxidized sediment was removed and underlying sediment depth of approximately 5 to 25 cm was collected with a shovel into 20 l plastic buckets, which were sealed with no headspace, and transported back to the laboratory. Sediments was stored at 15°C.

### Sediment incubations and current production

**Figure 2:**
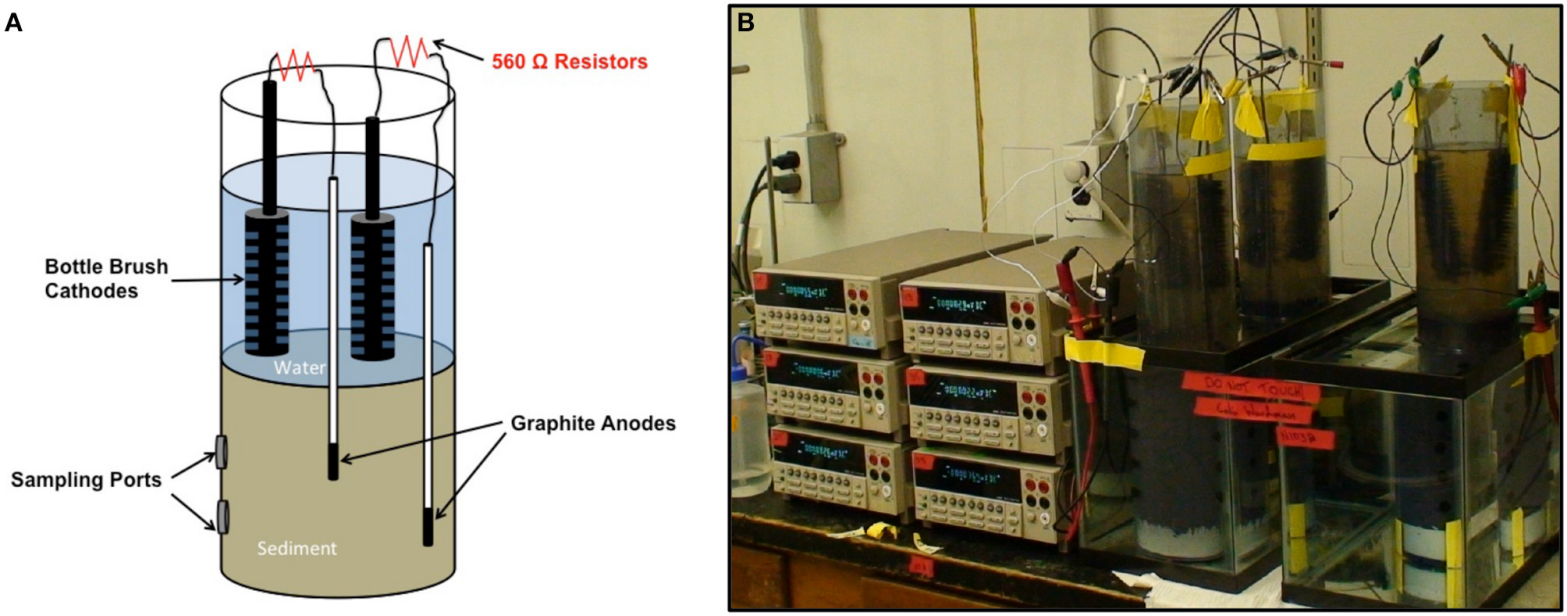
**Current monitoring approach**. **(A)** Schematic of sediment incubation cylinders. **(B)** Image of sediment incubations with current monitoring with digital multimeters.

Sediments were homogenized under a stream of N_2_ in a 120 l polyethylene container, fitted with a plastic top seal. The homogenized sediments were poured into PVC cylinders of either 7.6 cm diameter (Fe(III)-reducing sediments) or 10.2 cm diameter (sulfate-reducting or methanogenic sediments) that were sealed at the bottom with a butyl rubber stopper or PVC end caps (Figure 2). The sediment height was 23 cm. Water from the respective sites was poured on top of the sediments to provide 23 cm of standing water above the sediment. There were holes (10.5 mm diameter) in the sides of the PVC cylinders, sealed with butyl rubber stoppers to provide ports for subsampling the sediments for [2-^14^C]-acetate turnover studies (Figure 2).

The anodes were a graphite rod that sealed within a polystyrene pipet with marine epoxy such that just the end of the anode was exposed to the sediment, providing an accessible anode surface area of 28.26 mm^2^ (Figure 2). A marine-grade insulated wire was epoxied onto the anode and connected through a 560 Ω resistor to a bottle brush carbon cathode (length, 12.3 cm; width, 2.7 cm). Two anode assemblies were inserted into each sediment column, either 8 or 16 cm from the bottom of the cylinder. The two cathodes were placed such that the entirety of the brush was in the water above the sediment without touching the other cathode. Triplicate cylinders were placed in temperature-controlled chambers with the cylinders submerged in water-filled aquaria. The sediments were incubated at a range of temperatures to provide a range of rates of microbial metabolism for each sediment type. Although the experimental design was to provide six electrodes per temperature per sediment type, a number of the anode assemblies were faulty and either did not produce current or developed cracks in the pipets, which allowed microbial access to a greater surface area of the graphite than in the intact anode assemblies. Therefore, depending upon the integrity of the anode assemblies, 3–6 reliable current estimates were obtained for each incubation temperature with each sediment type.

Current production in the methanogenic sediments was monitored with either a Keithley 2700 or 2000 Digital Multimeter (Cleveland, OH) at hourly intervals. For the Fe(III)-reducing and sulfate-reducing sediments currents were monitored with a UEI DM284 Digital Multimeter (Beaverton, OR) on a daily basis.

### Acetate turnover rates

Once current densities reached a steady state for 4–10 days sediments from the same depth as the exposed surface of the anode were sampled through the side ports with a 3 cm plastic syringe with the distal end cut off. The sediment subsamples were immediately extruded under anoxic conditions into pre-weighed 60 ml serum bottles that were then sealed with a thick butyl rubber stopper. The weight of the added sediment was determined and the sediments incubated in a water bath at the temperature at which the sediments had previously been incubated. A anoxic solution (0.1 ml) of [2-^14^C]-acetate (American Radiolabeled Chemicals, Inc. St. Louis, MO; Specific Actvity, 45 mCi/mmol; Purity, 99%) was injected into the sediments to provide 1.2–1.7 μCi. This added ca. 15 μM acetate to the sediment pore water.

Over time 0.5 ml of headspace was sampled with a syringe and needle and injected into a gas chromatograph (model GC-8A,Shimadzu, Kyoto, Japan) connected to a GC-RAM radioactivity detector (LabLogic Broomhill, UK) to determine the quantity of ^14^CH_4_ and ^14^CO_2_ produced as previously described (Hayes et al., 1999). The first order rate constants for acetate metabolism in each sample were calculated from the initial linear rate of ^14^CH_4_ and ^14^CO_2_production according to *k* = *f* / *t* where *f* is the fraction of added label metabolized to product over an incubation time of *t*.

## Results and discussion

In order to determine whether the current produced at anodes emplaced in sediments could be correlated with rates of microbial metabolism at that location in the sediments, current production was compared with the rate of acetate mineralization. Acetate was chosen because it is the central intermediate in the anaerobic degradation of organic matter in sediments regardless of whether Fe(III) reduction, sulfate reduction, or methane production is the predominant terminal electron-accepting process (Lovley and Chapelle, 1995). Therefore, rates of acetate metabolism in these types of anoxic sediments is directly related to the overall rates that fermentable organic matter is being converted to carbon dioxide and methane.

It was hypothesized that current (I) would be directly related to the rate of acetate metabolism (V_a_), according to:

(reaction 1) $$\text{I}=\text{Z}\times {\text{V}}_{\text{a}}$$

where Z is a correlation constant which is the sum of what may be a substantial number of complex factors controlling how much current is produced in the sediments. An understanding the many complex factors that may contribute to the Z term is not necessary in order to use current production as a proxy for microbial metabolism as long as Z is constant over the range of conditions evaluated (i.e., there is strong direct correlation between I and V_a_).

Typically rates of acetate metabolism (V_a_) are estimated from the first order rate constant of the metabolism of radiolabelled acetate (k) and the concentration of acetate (A) where

(reaction 2) $${\text{V}}_{\text{a}}=\text{k}\times \text{A}$$

However, acetate concentrations in all three sediment types were below our detection limit of 10 μM with high performance liquid chromatography, preventing calculation of V_a_. This added another unknown and combining reactions 1 and 2:

(reaction 3) I=Z×k×A

At steady state, acetate concentrations acetate concentrations are controlled by the affinity of the microorganisms consuming the acetate and thus acetate concentrations are expected to be similar in sediments in which the same terminal electron-accepting predominates (Lovley and Chapelle, 1995). Therefore, within sediments with the same terminal electron-accepting process A can be considered a constant and, if the hypothesis of a direct correlation between current production and acetate metabolism holds, then there will be a direct correlation between current and the first order rate constant for acetate metabolism with the product of the two constants Z and A as the correlation constant:

(reaction 4) I=(ZA) × k

**Figure 3:**
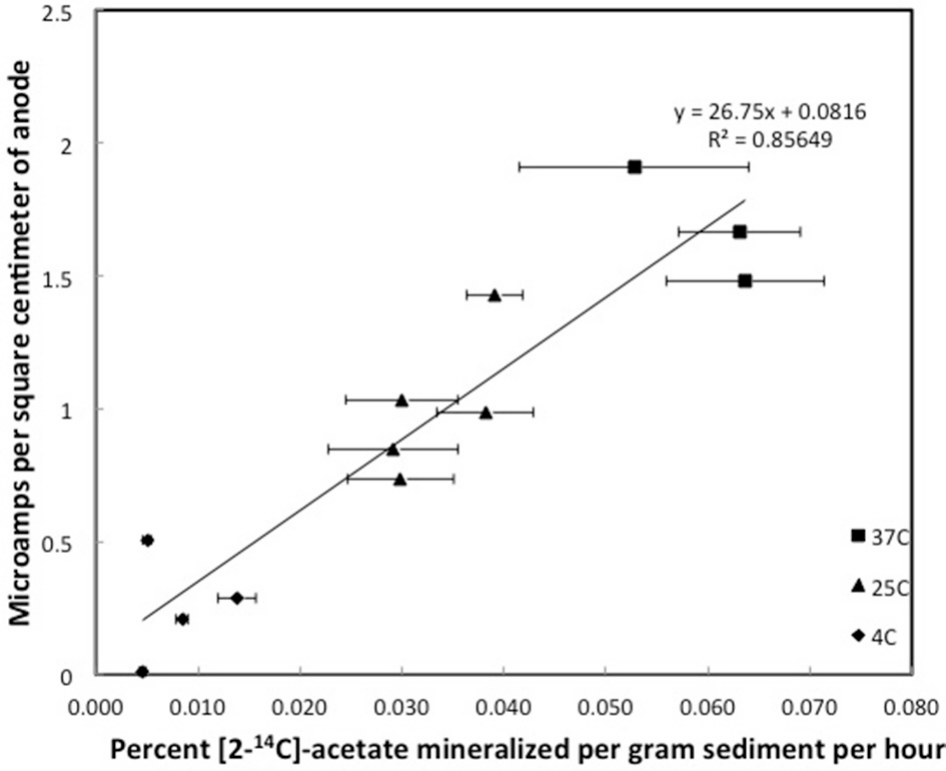
**Steady state currents and [2-^14^C]-acetate turnover rates in columns of methanogenic sediments**. Error bars represent the standard deviation of the mean for the mineralization of [2-^14^C]-acetate in triplicate incubations of sediment subsampled from the depth that the currents were recorded.

**Figure 4:**
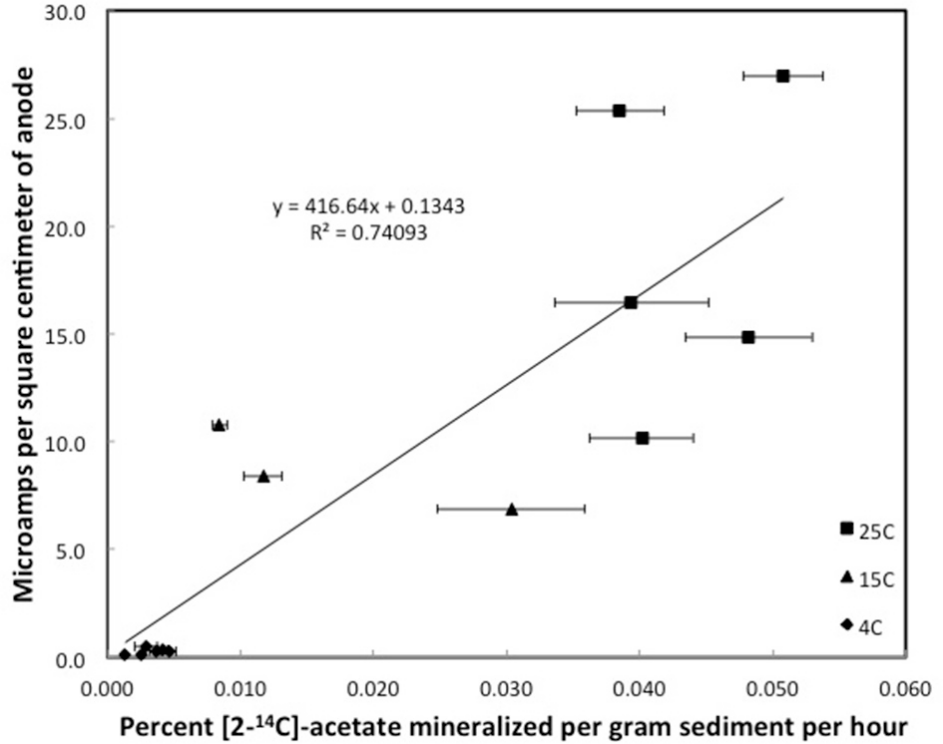
**Steady state currents and [2-^14^C]-acetate turnover rates in columns of sulfate-reducing sediments**. Error bars represent the standard deviation of the mean for the mineralization of [2-^14^C]-acetate in triplicate incubations of sediment subsampled from the depth that the currents were recorded.

**Figure 5:**
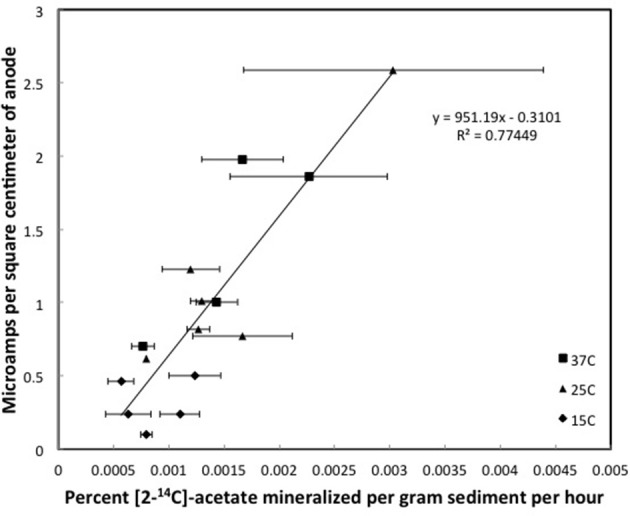
**Steady state currents and [2-^14^C]-acetate turnover rates in columns of Fe(III)-reducing sediments**, Error bars represent the standard deviation of the mean for the mineralization of [2-^14^C]-acetate in triplicate incubations of sediment subsampled from the depth that the currents were recorded.

In fact, there was a direct correlation between the first order rate constant for acetate metabolism and current produced in all three sediment types investigated (Figures 3–5). As expected, the rate constants for acetate metabolism in the subsurface sediments from the Rifle, CO site were much lower than for the freshwater or marine surface sediments, reflecting the higher organic content of the two surface sediments. With all sediments, incubation at different temperatures was an effective method for providing a range of different rates of microbial metabolism in each sediment type.

Although the acetate rate constants in the freshwater sediments in which methane production predominated and the marine sediments in which sulfate reduction predominated were similar, the currents produced in the marine sediments for comparable acetate turnover times were ca. 15-fold higher (Figures 3, 4), suggesting that the factor ZA was ca. 15-fold larger in the sulfate-reducing sediments. The higher ZA term for the sulfate-reducing sediments can not be attributed to higher acetate concentrations. Sulfate reducers have a higher affinity for acetate than methanogens (Lovley and Klug, 1983, 1986), thus the acetate pool is expected to be lower in sediments in which sulfate reduction predominates. In fact acetate measurements in sediments similar to those studied here revealed that the acetate pool in methanogenic sediments was twice as high as in sulfate-reducing sediments (Lovley and Phillips, 1987). This suggests that one or more of the many factors contributing to Z was greater in the sediments in which sulfate reduction was the terminal electron-accepting process.

One possibility is that there was an additional source of electron donor for current production in the sulfate-reducing sediments that was not available in the methanogenic sediments. In both sediment types, the production of acetate, as well as H_2_ and minor fermentation acids, near the anode surface is expected to supply electron donors for current production (Figure 1). At distance from the anode these electron donors support the reduction of sulfate or the production of methane. Methane is highly unreactive and is not likely to abiotically interact with the anode or to serve as an electron donor for microbially catalyzed current production. However, as noted in the Introduction, sulfide produced from sulfate reduction is highly reactive and is abiotically oxidized to elemental sulfur at anode surfaces (Tender et al., 2002; Gong et al., 2013). A diversity of microbes (Holmes et al., 2004; Zhang et al., 2014) can oxidize the elemental sulfur to sulfate with further current production (Figure 1). Therefore, microbial metabolism at greater distances from the anode can be captured as current production in marine sediments than is possible in methanogenic sediments.

These considerations suggest that although there is a direct correlation between current production and microbial activity in sediments in which methane production or sulfate reduction is the predominant terminal electron-accepting process, a different calibration will be needed to infer rates of microbial activity from specific current levels in the two types of sediments. Therefore, measurements of dissolved H_2_, or some other technique to determine the predominant terminal electron-accepting process will be important when interpreting current outputs to monitor microbial activity in environments in which there can be shifts between sulfate reduction and methane production.

In the Fe(III)-reducing sediments currents were more comparable to those in the sulfate-reducing sediments at similar acetate-turnover rates, and much higher than in the methanogenic sediments. As in the sulfate-reducing environments, microbial activity at distance from the anode in Fe(III)-reducing sediments may be reflected in current production at the anode because Fe(II) produced from Fe(III) reduction can diffuse to the anode and donate electrons (Figure 1).

## Implications

The results demonstrate that there are strong correlations between the current output of a simple anode-resistor-cathode device and rates of anaerobic microbial activity in a diversity of anoxic sediments. This is the first example of monitoring the *in situ* microbial activity in soils and sediments with a simple system that does not employ a poised anode and the first study to directly compare current production rates with an independent estimate of the rates of microbial activity.

It is expected that this technology will have broad application in the real-time monitoring of anaerobic microbial activity in a diversity of submerged soils as well as sediments. It offers the possibility of continuous monitoring of microbial activity over time without disturbing the soils/sediments. The small size of the anodes and low cost of the materials makes it feasible to study heterogeneities in microbial activity at multiple scales both horizontally and vertically. At the present stage of development, this SMART (*S*ubsurface *M*icrobial *A*ctivity in *R*eal *T*ime) technology will primarily be useful for monitoring relative changes in microbial activity in response to environmental perturbations, such as the response to temperature change shown here. However, other applications, such as deploying electrodes at the periphery of polluted sites as a sentinel to detect the migration of organic contaminants, are under investigation.

### Conflict of interest statement

The authors declare that the research was conducted in the absence of any commercial or financial relationships that could be construed as a potential conflict of interest.
